# Variability in climate change simulations affects needed long-term riverine nutrient reductions for the Baltic Sea

**DOI:** 10.1007/s13280-015-0657-5

**Published:** 2015-05-28

**Authors:** Arvid Bring, Peter Rogberg, Georgia Destouni

**Affiliations:** Water Systems Analysis Group, Institute for the Study of the Earth, Oceans and Space, University of New Hampshire, 8 College Road, Durham, NH 03824 USA; Department of Physical Geography and Quaternary Geology, Stockholm University, 106 91 Stockholm, Sweden

**Keywords:** Baltic Sea drainage basin, Baltic Sea Action Plan, Climate change, Waterborne nutrient transport, CMIP5 general circulation models, Runoff projections

## Abstract

Changes to runoff due to climate change may influence management of nutrient loading to the sea. Assuming unchanged river nutrient concentrations, we evaluate the effects of changing runoff on commitments to nutrient reductions under the Baltic Sea Action Plan. For several countries, climate projections point to large variability in load changes in relation to reduction targets. These changes either increase loads, making the target more difficult to reach, or decrease them, leading instead to a full achievement of the target. The impact of variability in climate projections varies with the size of the reduction target and is larger for countries with more limited commitments. In the end, a number of focused actions are needed to manage the effects of climate change on nutrient loads: reducing uncertainty in climate projections, deciding on frameworks to identify best performing models with respect to land surface hydrology, and increasing efforts at sustained monitoring of water flow changes.

**Electronic supplementary material:**

The online version of this article (doi:10.1007/s13280-015-0657-5) contains supplementary material, which is available to authorized users.

## Introduction

Eutrophication of marine waters is a growing problem in many regions of the world (Hallegraeff 1993; Rabalais et al. 2009). In response to increasing nutrient loads and associated algal blooms, hypoxia, i.e., dead zones, is also expanding (Diaz 2001) and has now been documented in over 400 marine systems globally (Diaz and Rosenberg 2008). The Baltic Sea has for several decades been subject to severe eutrophication and increasing hypoxia (Karlson et al. 2002; Carstensen et al. 2014). This situation has come about due to a number of factors, including low inflow of salt water through the Danish Straits, nutrient leaching from agricultural activities in the basin, wastewater and water treatment plants, and direct atmospheric deposition on the sea (Conley et al. 2009).

Under the Helsinki Commission (HELCOM), states around the Baltic Sea have agreed to reduction targets to their respective nutrient loads in order to address this problem. Nutrient reduction targets form a key component of the Baltic Sea Action Plan (BSAP), agreed to in 2007 by the nine countries with coastline on the sea. According to the plan, countries have to remove specific amounts of nitrogen and phosphorus from their respective loadings to the sea. These reduction amounts were recently revisited and updated and are now termed Country Allocated Reduction Targets (CARTs; http://helcom.fi/baltic-sea-action-plan/nutrient-reduction-scheme/targets).

The BSAP constitutes a medium-term agenda for addressing eutrophication problems, with targeted reductions “aiming at reaching good ecological and environmental status by 2021” (HELCOM 2007). At the same time, long-term climate change is already evident in the Baltic, for example, through increasing as well as decreasing runoff in various parts of the region (HELCOM 2013a). These and other related processes will have a growing impact in coming decades (HELCOM 2011, 2013a). Depending on how they evolve in the future, they may also interfere with Baltic states’ ability to meet the BSAP targets, or risk undermining the enduring value of actions under the BSAP (HELCOM 2013a).

Several recent studies have investigated projected climate change scenarios in attempts to determine future riverine nutrient loads to the Baltic (see also Andersson et al. 2015), but some results are inconclusive. For example, two recent studies have used data from a small number of global climate models (three or four), downscaled through a regional climate model, whose output then in turn drives a hydrological model (Arheimer et al. 2012; Hägg et al. 2014) to simulate runoff and associated nutrient load changes. While results reported in Arheimer et al. (2012) indicate overall reductions in nitrogen loads to the Baltic due to climate change, Hägg et al. (2014) found increases in nitrogen loads for all the climate scenarios they studied.

Increasing nutrient loads were also found in a study by Meier et al. (2012), in which no hydrological model was used. That study was instead centered on an ocean model, with runoff into the Baltic Sea estimated from the water balance obtained for the Baltic Sea drainage basin by regional climate modeling. Nutrient loads were then estimated assuming that concentrations in surface waters would not change, as found to have been the case historically in a study by Stålnacke et al. (1999).

The approach of downscaling global model output to a regional model allows for the use of dedicated hydrological modeling to study climate change effects on runoff. The downscaling itself is usually achieved using either statistical or dynamical procedures, or some combination of the two. The former approach seeks to statistically correlate finer resolved patterns in spatial data with the coarser output of global models. The latter makes use of a medium-scale regional model (often at continental scale) that takes as its input the output data of a coarser resolved, global model.

However, any climate change-related output of the hydrological model still fundamentally depends on the input data from the global climate model used, as shown by, e.g., Arheimer et al. (2012). Therefore, at least for relatively large drainage basins, the output of global climate models can also be studied directly to investigate projected changes to runoff (Jarsjö et al. 2012; Bring and Destouni 2014).

The above-mentioned studies of climate change effects on nutrient loads into the Baltic Sea (Arheimer et al. 2012; Meier et al. 2012; Hägg et al. 2014) were based on versions of the ECHAM5 and HadCM3 global climate models to provide climate scenarios, with the CCSM model also considered in the study by Hägg et al. (2014). However, other studies report large inter-model variances when comparing the outputs of (or output implications for) hydrological variables from multiple global (Jarsjö et al. 2012; Bring and Destouni 2014) and regional (Teutschbein et al. 2011; van der Velde et al. 2013) climate models, including such from different model generations. The Baltic Sea load studies (Arheimer et al. 2012; Meier et al. 2012; Hägg et al. 2014) were also all based on emission scenarios from the Intergovernmental Panel on Climate Change (IPCC) Special Report on Emission Scenarios (SRES; Nakicenovic and Swart 2000), which has now been superseded by Representative Concentration Pathways (RCPs; Moss et al. 2010). There is thus a need for investigating the impact of variability in a set of climate models and of the new RCP considerations and generation of climate models used by the IPCC, for climate change projection effects on the freshwater runoff and the associated waterborne nutrient loads to the Baltic Sea.

In this paper, we investigate the medium- and long-term effects of climate change on runoff to the Baltic Sea, through the impact of the variability of a set of state-of-the-art global climate models and RCP scenarios from the most recent Coupled Model Intercomparison Project (Phase 5; CMIP5). We further investigate the impact of these runoff changes on nutrient loads, assuming unchanged nutrient concentrations, determined as part of the HELCOM monitoring and reporting process. We finally also compare the climate-driven nutrient load changes and the model variability around such changes with the recently updated load reductions that have been committed to under the BSAP (HELCOM 2013b).

We emphasize that this study focuses solely on the direct climate change effect, which will influence both runoff and nutrient loads irrespective of other anthropogenic changes that may concurrently take place. Our aim with this scenario study is not to provide exact estimates of climate change, nor any precise appraisal of the ensuing load changes. Rather, we aim to investigate the effects of a wider set of climate model outcomes on riverine nutrient loads that we consider equally likely within the context of this study.

## Materials and methods

**Fig. 1:**
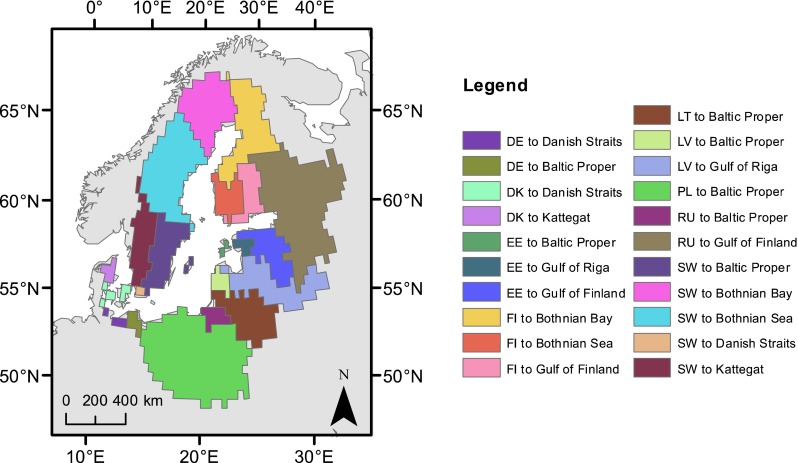
Map of the Baltic Sea drainage basin showing the 21 basins used to delineate climate model results

We used the comprehensive watershed delineation of the Baltic Sea drainage basin presented in Hannerz and Destouni (2006) and re-gridded it from 1 km to 0.5 degree spatial resolution for this large-scale climate application. This resolution is common in large-scale hydrological applications and retains all details of climate model output as it is finer than the grids of that data. The whole contributing drainage basin area was further divided into sub-basins, specific to each country part in each main marine basin receiving nutrient loads from land. This led to a total of 21 sub-basin combinations within the Baltic Sea drainage basin (Fig. 1).

The World Climate Research Programme’s CMIP5 project makes available a wide set of data generated by global climate models. The CMIP5 framework and protocols ensure that output generated within the scope of the project is comparable across models and adheres to the same standards (Taylor et al. 2012). Data generated within the project are freely available and constitutes the most up-to-date and authoritative set of climate model projections for both historical and future scenarios.

To analyze CMIP5 projections for the 21 basins, we downloaded data for all models that provided data for surface runoff (termed *total runoff* and abbreviated as *mrro* in CMIP5 datasets), for the historical, RCP2.6 and RCP8.5 experiments, from the PDCMI data portal for CMIP5 (PCDMI 2013). We here included all possible CMIP5 climate model data, instead of selecting a particular model or a few models. We used one realization from each model with equal initial conditions and boundary conditions. Primarily, we therefore addressed model variability, which particularly on longer time horizons represents a large portion of climate uncertainty (Fig. 11.8f in Kirtman et al. 2013). This way, we sampled the space of model configurations and included the source of uncertainty that arises from various representations of physics.

The historical experiment attempts to reproduce the observed evolution of the climate system. The RCP2.6 experiment corresponds to an optimistic scenario of rapid CO_2_ emission reductions, while the RCP8.5 experiment corresponds to a high-emission, business-as-usual scenario. Table 1 shows some further details of the two scenarios.

**Table 1:** Details of the RCP2.6 and RCP8.5 emission scenarios

	RCP2.6	RCP8.5
Emission pathway during 2000–2100	Peaking in 2050, then declining	Increasing
Radiative forcing in 2100 (W m^−2^)	2.6	8.5
CO2 concentration in 2100 (ppm)	420	935

We further re-gridded all climate model data to coincide with the 0.5 degree resolution of the drainage basin outlines and computed area-weighted averages of CMIP5 model output over each of the 21 basins. In this step, not all models provided runoff data for a geographical domain that coincided fully with all the delineated basins around the Baltic Sea. The 13 models that did, and were finally included in the present study, are listed in Supplementary Material (S), Table S1. In the following, any reference to the *ensemble* of models refers to this particular set and not to the CMIP5 ensemble in its entirety.

With any selection of a subset of an ensemble, there is a risk of introducing bias pertaining to that particular subset of models. Although we cannot exclude that biases exist for the models we studied, they perform on average as the full CMIP5 ensemble when evaluated with regard to the global seasonal-cycle climatology (Fig. 9.7 in Flato et al. 2013).

From the model output, we aggregated runoff values over three 30-year time periods of 1961–1990 (historical experiment), 2010–2039, and 2070–2099 (RCP2.6 and RCP8.5 experiments) and calculated changes for the two latter periods, using the historical period as a reference. To estimate values per country, we averaged runoff values for basin polygons within the same country and also calculated total flow values per country by multiplying with combined polygon area. Figure S1a shows the modeled historical values of total flow and the flow values reported by HELCOM.

We subsequently also calculated nutrient load changes from climate effects as Δ*L*_clim_ = *C* × Δ*Q*, where Δ*L*_clim_ is the total nutrient load change from a basin, C is the average nutrient concentration in river water, and Δ*Q* is the modeled flow change from the basin. Our approach here follows that of Meier et al. (2012) in using the observation by Stålnacke et al. (1999) of near-constant nutrient loads to the Baltic Sea when loads were normalized against discharge. This observation is equivalent to near-constant riverine concentrations of total nitrogen and total phosphorus over several decades, despite any population and other anthropogenic change in the basin. Indeed, this is somewhat counter-intuitive, particularly since total nitrogen and total phosphorus represent mainly dissolved and mainly particulate fluxes, respectively, and these could be expected to behave differently. Therefore, an implication is that there could be important shifts in the bioavailability of nitrogen and phosphorus (and hence ratios of plant-available nutrients in aquatic systems) with changing climate regimes that are not captured here. Nevertheless, the observation was confirmed for 14 Baltic basins, and also reported for another 21 large basins in the Mississippi–Atchafalaya drainage, by Basu et al. (2010). There are also other reports of runoff as the predominant control on nutrient export (Morse and Wollheim 2014).

The relationship reported by Basu et al. (2010) was stronger for nitrogen than for phosphorus and also mainly applicable to nutrient-rich and extensively managed basins. For the Baltic Sea, the majority of loads arise in extensively managed basins. A potential explanation was put forth by Basu et al. (2010), who see evidence of emergent biogeochemical stationarity. This means that present loads are buffered by a legacy of stored nutrients, which were applied earlier but partly still remain in the landscape and there act to reduce the variability in concentrations. The effect is similar to that of certain geogenic constituents that also show a linear relationship between total load and discharge. Thus, we consider the relatively small temporal changes (and change trends) to nutrient concentrations in surface water over larger areas reported over long time periods by both Stålnacke et al. (1999) and Basu et al. (2010) as a reasonable baseline also for future conditions in the Baltic region. We recognize, however, that future anthropogenic changes may alter the situation, in ways not yet certain, and also depending on the magnitude of change.

In the absence of clearly defined scenarios for changes in nutrient concentration consistent with the agreed-upon land-use and population changes driving the greenhouse gas emission scenarios of the RCPs, we limited our study to the effects of climate change and river discharge and associated nutrient fluxes, assuming current nutrient concentration levels. In this way, our results can be interpreted as pertaining specifically to hydrological effects of large-scale climate drivers over the Baltic. These climate drivers constitute a force that is beyond the sole control of the population in the region.

The long-term average nutrient concentrations C were determined as *C* = *L*/*Q*, where *L* and *Q* are annual average values of reported nutrient loads and water discharges, respectively, from each basin into the Baltic Sea (HELCOM 2013b^1^). Resulting long-term average concentration levels of total nitrogen and total phosphorus in surface waters for each basin and country are listed in Table 2. Figure S1b, c shows the total loads calculated from these concentrations and the historical modeled runoff, compared with the loads reported by HELCOM. Finally, each country’s climate-driven nutrient load changes Δ*L*_clim_ were aggregated as a sum of its individual basin Δ*L*_clim_ values.

**Table 2:** Nutrient concentrations in surface waters, based on reported flows and loads for each country’s contributing basins in the Review of the Fifth Baltic Sea Pollution Load Compilation for the 2013 HELCOM Ministerial Meeting (HELCOM 2013b). Concentrations were calculated as *C* = *L*/*Q* for annually reported values of *L* and *Q* for each basin. The 1994–2010 average value is shown. For countries with multiple basins, the totals are calculated in the same way but based on sums of flows and loads for all basins. Numbers in brackets are coefficients of variation. Country totals (whether aggregated from multiple sea basins or not) are listed in bold

Country	Basin	Total nitrogen		Total phosphorus	
		Concentration in surface waters (mg l^−1^)	Load (tons year^−1^)	Concentration in surface waters (mg l^−1^)	Load (tons year^−1^)
DE	Baltic Proper	4.66 (0.24)	7528	0.12 (0.15)	184
DE	Western Baltic	5.17 (0.16)	12 310	0.15 (0.10)	336
**DE**	**Total**	**4.96 (0.17)**	**19 838**	**0.14 (0.10)**	**520**
DK	Baltic Proper	6.22 (0.14)	1837	0.18 (0.06)	51
DK	Kattegat	4.81 (0.11)	23 738	0.15 (0.07)	757
DK	Sound	4.35 (0.17)	1561	0.30 (0.18)	105
DK	Western Baltic	5.89 (0.16)	20 181	0.20 (0.12)	659
**DK**	**Total**	**5.24 (0.14)**	**47 318**	**0.18 (0.08)**	**1572**
EE	Baltic Proper	2.21 (0.23)	990	0.05 (0.27)	22
EE	Gulf of Finland	1.58 (0.14)	11 180	0.06 (0.19)	423
EE	Gulf of Riga	2.28 (0.17)	11 662	0.05 (0.24)	265
**EE**	**Total**	**1.88 (0.12)**	**23 832**	**0.06 (0.17)**	**710**
FI	Archipelago Sea	2.51 (0.34)	6784	0.12 (0.24)	332
FI	Bothnian Bay	0.61 (0.11)	31 193	0.03 (0.11)	1561
FI	Bothnian Sea	1.42 (0.16)	16 225	0.06 (0.15)	644
FI	Gulf of Finland	1.05 (0.14)	13 772	0.04 (0.17)	566
**FI**	**Total**	**0.87 (0.12)**	**67 974**	**0.04 (0.11)**	**3103**
**LT**	**Baltic Proper**	**2.19 (0.34)**	**46 096**	**0.11 (0.25)**	**2313**
LV	Baltic	2.50 (0.23)	11 152	0.06 (0.30)	284
LV	Gulf of Riga	2.31 (0.23)	66 448	0.07 (0.28)	1963
**LV**	**Total**	**2.33 (0.21)**	**77 600**	**0.07 (0.26)**	**2247**
**PL**	**Baltic Proper**	**3.23 (0.14)**	**202 775**	**0.20 (0.12)**	**12 228**
RU	Baltic Proper	2.01 (0.21)	8800	0.15 (0.21)	660
RU	Gulf of Finland	0.77 (0.05)	65 853	0.06 (0.15)	4990
**RU**	**Total**	**0.83 (0.06)**	**74 653**	**0.06 (0.15)**	**5650**
SW	Baltic Proper	1.43 (0.09)	27 154	0.04 (0.11)	698
SW	Bothnian Bay	0.29 (0.09)	15 779	0.02 (0.23)	803
SW	Bothnian Sea	0.33 (0.06)	25 808	0.01 (0.23)	889
SW	Kattegat	1.10 (0.11)	31 952	0.02 (0.16)	688
SW	Sound	5.69 (0.17)	4544	0.10 (0.13)	77
**SW**	**Total**	**0.58 (0.10)**	**105 237**	**0.02 (0.13)**	**3155**

In order to evaluate the effect of climate change projections on the countries’ reduction targets under the BSAP, we calculated the per-country total required load reduction, Δ*L*_req_. We define Δ*L*_req_ = Δ*L*_BSAP_ + Δ*L*_clim_ as a country’s total required reduction in nutrient load, when accounting for both the country’s BSAP reduction target (Δ*L*_BSAP_) and any additional effect of the modeled climate change (Δ*L*_clim_). The additional effect of climate change may either increase the needed reduction (to >Δ*L*_BSAP_), in the case of a climate-projected load increase (positive Δ*L*_clim_), or decrease it (to <Δ*L*_BSAP_), in the case of a climate-projected background decrease in loads (negative Δ*L*_clim_).

In order to facilitate direct comparisons between countries, we also normalized Δ*L*_req_ by each country’s reduction target according to the BSAP, to the relative measure:

$$ \Delta L_{\text{req - rel}} = \, (\Delta L_{\text{BSAP}} + \Delta L_{\text{clim}} )/ \Delta L_{\text{BSAP}} , $$

where Δ*L*_BSAP_ is the country’s reduction target in the BSAP and Δ*L*_clim_ is the change in nutrient load arising from climate change projections. For example, a reduction target of 10 000 tons and an added 2000 tons of expected climate-driven load change will yield a total required load reduction Δ*L*_req-rel_ = 1.2, or 120 %, relative to the country’s BSAP target, which serves as a reference (factor 1.0 or 100 %). The values of Δ*L*_BSAP_ are listed in Table 3, along with the results of Δ*L*_req_ and Δ*L*_clim_ that are discussed further in the following.

**Table 3:** Values of annual load reductions required under the BSAP (Δ*L*_BSAP_), load reductions or increases arising from climate change (Δ*L*_clim_), and resulting total needed load reductions (Δ*L*_req_). Δ*L*_BSAP_ values are updated figures based on the Review of the Fifth Baltic Sea Pollution Load Compilation (HELCOM 2013b). All values are in tons year^−1^

Country	Δ*L*_BSAP_	RCP2.6				RCP8.5			
		2010–2039		2070–2099		2010–2039		2070–2099	
		Δ*L*_clim_	Δ*L*_req_	Δ*L*_clim_	Δ*L*_req_	Δ*L*_clim_	Δ*L*_req_	Δ*L*_clim_	Δ*L*_req_
Nitrogen									
DE	1953	748	2701	1398	3351	556	2509	5279	7232
DK	971	308	1279	543	1514	−332	639	4170	5141
EE	1584	145	1729	−506	1078	−1378	206	1643	3227
FI	2492	−741	1751	−881	1611	−3004	−512	−4402	−1910
LT	8428	981	9409	939	9367	652	9080	7239	15 667
LV	1439	1367	2806	772	2211	292	1731	9445	10 884
PL	39 257	7713	46 970	12 978	52 235	9477	48 734	44 632	83 889
RU	9356	−971	8385	−654	8702	−2609	6747	−3725	5631
SW	7477	−189	7288	−465	7012	−1590	5887	−1101	6376
Phosphorus									
DE	170	19	189	36	206	14	184	136	306
DK	38	16	54	30	68	−14	24	215	253
EE	320	5	325	−17	303	−47	273	58	378
FI	356	−34	322	−37	319	−130	226	−202	154
LT	1470	49	1519	47	1517	33	1503	363	1833
LV	220	40	260	23	243	10	230	276	496
PL	7480	465	7945	783	8263	571	8051	2691	10 171
RU	3790	−74	3716	−50	3740	−198	3592	−283	3507
SW	530	−12	518	−14	516	−52	478	−97	433

## Results

**Fig. 2:**
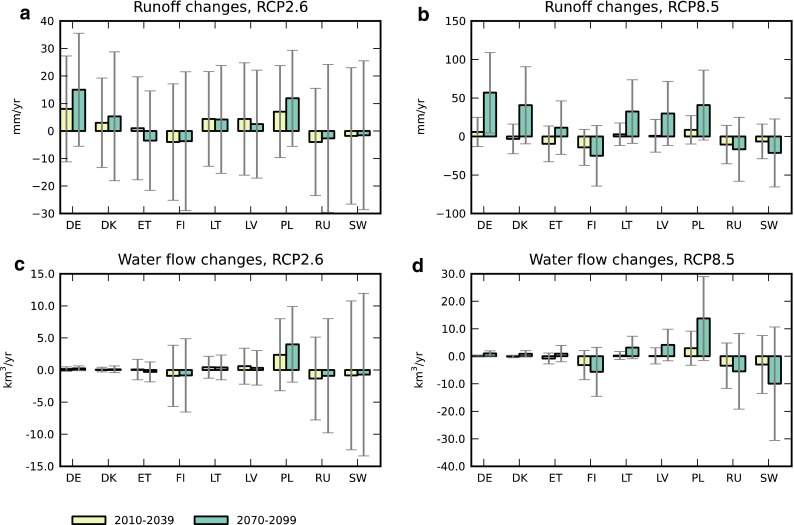
Mean model (*N* = 13) changes to **a**, **b** runoff and **c**, **d** volumetric water flows for nine countries in the BSDB, from the period 1961–1990 to future periods 2010–2039 and 2070–2099, and for emission scenarios RCP2.6 (**a**, **c**) and RCP8.5 (**b**, **d**). *Error bars* denote one standard deviation of individual model values

Runoff change projections of the CMIP5 ensemble subset generally vary greatly among different models (Fig. 2). For most countries, and in particular for the near-term period of 2010–2039, the standard deviation of model change is several times the size of the ensemble mean change. This implies a very large spread in model projections of future runoff changes, although for Poland and Germany the ensemble signal is slightly more pronounced in relation to the inter-model variability (Fig. 2a). For the long-term projections in the high-emission scenario, the magnitude of the ensemble signal is more pronounced for all countries, albeit smaller for Estonia, Russia, and Sweden (Fig. 2b).

The variability in model runoff per area [LT^−1^] is also reflected in projected changes to the volumetric water flows [L^3^T^−1^] (freshwater discharges to the sea), but a few countries with relatively large catchment areas then dominate absolute changes in the freshwater contribution to the Baltic Sea (Fig. 2c, d). A relatively strong ensemble signal of increasing water flow emerges particularly for Poland, and in the high-emission scenario also for Finland, Russia, and Sweden, although inter-model variability is higher for the latter countries.

This change in flow carries varying amounts of nutrients with it, depending on the concentrations of nitrogen and phosphorus in the surface waters of the respective basin (see reported concentrations in Table 2). Figure S2 shows the model-projected climate-driven change in nutrient load per surface area. Germany, Denmark, and Poland have the highest nutrient concentration in surface waters and are therefore also the countries with the highest projected nutrient load changes per unit area. In the long term, the drainage areas of Lithuania and Latvia should also exhibit greater phosphorus loading (Fig. S2b, c).

**Fig. 3:**
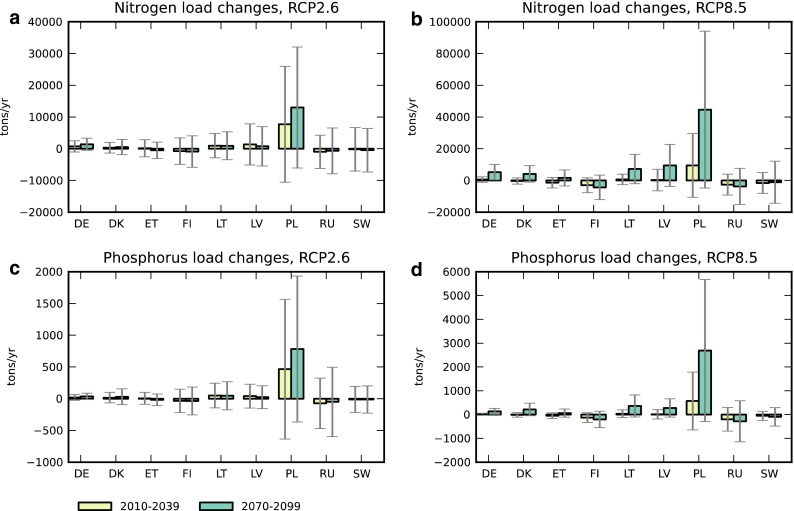
Mean model (*N* = 13) changes to **a**, **b** total N loads and **c**, **d** total P loads for nine countries in the BSDB, from the period 1961–1990 to future periods 2010–2039 and 2070–2099, and for emission scenarios RCP2.6 (**a**, **c**) and RCP8.5 (**b**, **d**). *Error bars* denote one standard deviation of individual model values

The total load change—when accumulated over the entire drainage basin of each country—is shown in Fig. 3. For nitrogen loads, the ensemble mean values indicate that Poland is subject to the largest absolute change, particularly over the long term in the high-emission scenario (Fig. 3b). The relatively small sizes of the German and Danish Baltic drainage areas lead to relatively small total loads for these countries. Uncertainty is also here very large, however, and for all countries, the standard deviation of projected changes is greater than the mean value. For phosphorus, the situation is similar (Fig. 3c, d).

**Fig. 4:**
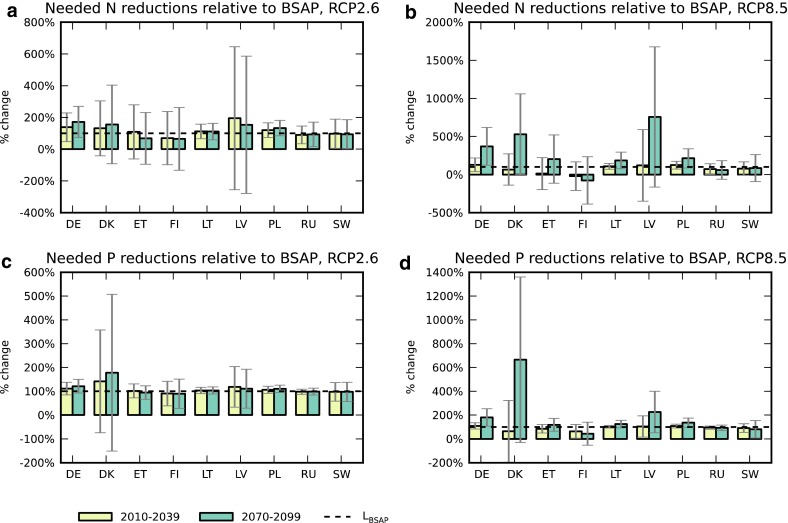
Required load reductions, Δ*L*_req-rel_ = (Δ*L*_BSAP_ + Δ*L*_clim_)/Δ*L*_BSAP_, relative to reduction targets under the Baltic Sea Action Plan (Δ*L*_BSAP_), and accounting also for climate-driven load changes (Δ*L*_clim_). *Error bars* denote one standard deviation of climate model-projected Δ*L*_clim_, normalized with each country’s Δ*L*_BSAP_

Our measure of normalized total required load reductions, in relation to commitments under the BSAP, shows that the effect of model variability in the climate-driven changes in nutrient loads varies greatly between countries and between nitrogen and phosphorus. For nitrogen, climate model changes have a standard deviation of several tens of percent for all countries (Fig. 4a, b). For phosphorus, climate model changes are much smaller for some countries, in particular for Russia (Fig. 4c, d). When accounting for model-projected climate change, the variability in the required total nitrogen reductions for Denmark and Latvia is up to an order of magnitude larger than the reductions now committed to under the BSAP (Fig. 4b). For Denmark, the same is true for phosphorus (Fig. 4d).

## Discussion

The large variability in runoff projections for the Baltic drainage is in line with multi-model projections presented by the IPCC for Northern Europe. For the relatively near term of 2016–2035 in particular, model mean changes are indicated to be less than a standard deviation of model variability (Figs. AI.38–AI.39 in van Oldenborgh et al. 2013). This model-projected variability in climate change effects on runoff, approximately of equal magnitude for all countries (Fig. 2), translates to variations in the signal strength of nutrient load changes, in relation to the noise of inter-model variation (Fig. 3). However, when put in the context of countries’ activities under the BSAP, the magnitude of the climate-driven change signal, and particularly the model variability in that signal, has vastly different impacts on various countries.

For all countries, but in particular Denmark and Latvia, and to a degree also Estonia and Finland, climate change projections add a major factor of variability to the task of reducing nitrogen under the BSAP. Some countries’ load reduction commitments are relatively small in relation to the potential effect of climate change. For those countries, climate change may lead to climate-driven load reductions that fully match or exceed the reductions outlined in the BSAP. Conversely, the reduction measures carried out to comply with the BSAP could just as well be entirely nullified due to climate-driven increase in loads, perhaps several times greater than any effect of human intervention. This situation arises even in the relatively near term and irrespective of the emission pathway that eventually becomes realized.

The ability to reach phosphorus reduction targets is also quite uncertain for Denmark and Latvia, and to a lesser extent for Finland and Sweden. In contrast to the case for nitrogen, “free” climate-driven achievement of phosphorus targets is less likely for most countries, as the variability in climate-driven load changes is generally smaller than for nitrogen. Climate model variability has a particularly small effect on the phosphorus reductions required for Russia, Poland, and Lithuania. It should be noted, however, that even small increases to required reductions may be relatively costly, depending on whether the economically best performing measures are selected first or not (Volk et al. 2008). Even if the least costly actions are rationally implemented first, only more expensive options are then left on the table for any additional reductions needed to reach target loads.

The temporal horizon of the BSAP is primarily the time until 2021, whereas the climate change effects we study here have been investigated for both 2010–2039 and the longer-term period of 2070–2099. However, as inter-annual variability around the long-term average is large for runoff, it is unlikely that the latter would exhibit step-wise change arriving abruptly some time in the future. Climate-driven runoff change thus constitutes a long-term trend effect that needs to be accounted for already in the time perspective of actions to achieve BSAP targets. While other change drivers, for example, changing land use, may also influence nutrient exports (Kaushal et al. 2008; Hale et al. 2013), climate will remain a background factor irrespective of such other changes. In particular, the large model variability in the climate-driven runoff and associated nutrient load changes is a strong call for two priorities. Firstly, a focus on continuous monitoring is needed, to follow up and discriminate which direction the runoff and load changes are in fact taking. Secondly, improved modeling of the land water system may yield reduced model variability and possibly provide more clear indications of change directions.

Our analysis constitutes a first-order assessment of the range of potential impacts that various future scenarios may have on the management of nutrient reductions for various countries. It should not be considered as an exact basis for detailed calculations of potential costs. In particular, we acknowledge limits to the analysis in terms of finer resolved dynamics of nutrient transport and in potential intra-seasonal variations of both nutrient fluxes and discharge change patterns. A more detailed study, incorporating also coupled nutrient transport modeling, would have to be carried out for investigating such effects. Although we anticipate that such a study might alter our conclusions for certain basins, we expect that the picture of radically different effects of climate on the various countries across the Baltic region would remain.

Concurrent with climate change, other changes may also affect the total loads on the Baltic Sea. Direct atmospheric deposition on the sea is one such effect, and a dramatically altered socioeconomic context another. In the end, management of anthropogenic nutrient loads to the environment must account for the demonstrated uncertainty in a relevant way. Environmental management targets in terms of fixed numbers, as the BSAP load reduction requirements, are tangible and provide a clear and transparent objective. However, it may prove more fruitful to explore adaptive governance measures that also incorporate uncertainty and continuous monitoring into the implementation and enforcement of load reduction measures (Mee 2005; see also Borgström et al. 2015; Nilsson and Bohman 2015). Although the numbers in the BSAP are acknowledged as provisional and have already been subject to revision by the contracting parties (HELCOM 2007, 2013b), it is likely that the entire eradication of a country’s targeted reduction from climate change causes was not envisioned when the original target numbers were agreed upon and would most likely not be accepted by other contracting parties that may need to still bear their commitments unchanged or even increased.

A principal question that needs to be answered is: Should BSAP targets be considered fulfilled, irrespective of whether country reductions have come about by human intervention or by climate change effects? Or, phrased differently: Should BSAP parties be held to their commitments to reduce their loads by a certain amount and, at high cost, irrespective of the actual load effects of the concurrent process of climate change?

In more general terms, such management-related questions may also arise in other contexts where actions aiming at restoring ecosystems involve changes to hydrological loads. For instance, the uncertainty introduced in the land water system’s response to climate change may also apply to other ecosystem-critical loads, such as the influx of silica, which is strongly affected by large-scale hydrological alterations on land and influences marine phytoplankton populations in coastal seas around the world (Ittekkot et al. 2000). Hypothetically, one could consider a kind of compensation scheme to continually evaluate and balance the uncertain effect of climate change on needed reduction commitments for the parties. Furthermore, the general question of reversibility from eutrophication, discussed for the Baltic Sea and other coastal ecosystems in Duarte et al. (2009), is likely also influenced by this uncertainty. A more reliable quantification of land water flow changes under climate change, and formalized treatment of its uncertainty, would contribute to more robust management actions for ecosystem restoration.

## Conclusion

Our study of a subset of the CMIP5 multi-model ensemble shows that uncertainty propagation from variability in climate modeling, through runoff projections, to water flow and nutrient load projections has widely varying consequences for the various parties to the BSAP. Some countries’ reduction allocations are relatively small in relation to the potential effect of climate change, and this means that nutrient load reductions tantamount to their entire commitments, and more, could be realized entirely through climate change. Equally likely, however, is that the human actions to comply with targets are entirely insufficient due to climate change effects, since these are so highly variable in the suite of climate models studied.

From the great span in the change implications of different climate models, it follows that a particular choice of model may critically affect the projection outcome for climate-driven changes of nutrient loads. Ideally, the model should be scientifically assessed and justified as a generally best performing and uncertainty-reducing model choice for the relevant hydro-climatic variables. Such choices are not straightforward, however.

A recent analysis for the pan-Arctic drainage basin, which extends over the same latitudes as the Baltic Sea drainage basin, indicated low correlation between models when ranked according to their ability to simulate temperature or precipitation (Bring and Destouni 2014). Selecting an overall best performing model in terms of thermodynamics (i.e., temperature) may therefore result in sub-optimal performance of precipitation and other water flux variables. Multi-model ensembles have also been shown to provide more accurate results than single models for hydrological applications (Foster and Uvo 2010). Furthermore, the study by Bring and Destouni (2014) also indicated that models may be right for the wrong reasons, as small bias errors in some cases resulted from large absolute errors canceling out over drainage basin scales.

Thus, agreement on which model(s) to use, and a framework to make such agreements, should be a priority for decisions on how to incorporate climate change effects in a long-term strategy for a viable Baltic Sea ecosystem status.

## Electronic supplementary material

Supplementary material 1 (PDF 288 kb)

## Footnotes

1 Full dataset available at http://helcom.fi/Documents/Baltic%20sea%20trends/Eutrophication/PLC-5.5%20dataset%20for%20web.xlsx.
