# Supplementary material for: Variability in climate change simulations affects needed long-term riverine nutrient reductions for the Baltic Sea
Source: Ambio. 2015 May 28;44(Suppl 3):381–91. doi: 10.1007/s13280-015-0657-5 (PMC4447692; doi:10.1007/s13280-015-0657-5)
Supplement: Supplementary file 1 — Supplementary material 1 (PDF 288 kb) [file 13280_2015_657_MOESM1_ESM.pdf]

AMBIO

Electronic Supplementary Material

This supplementary material has not been copy edited by the publisher or the editorial office.

Title: **Variability in climate change simulations affects needed long-term riverine nutrient reductions for the Baltic Sea**

Authors: Arvid Bring, Peter Rogberg, Georgia Destouni

Table S1 List of climate models included

Model Name	Institution
BCC-CSM1.1	Beijing Climate Center, China Meteorological Administration
BNU-ESM	College of Global Change and Earth System Science, Beijing Normal University
CanESM2	Canadian Centre for Climate Modelling and Analysis
CSIRO-Mk3.6.0	Commonwealth Scientific and Industrial Research Organization in collaboration with Queensland Climate Change Centre of Excellence
FGOALS-g2	LASG, Institute of Atmospheric Physics, Chinese Academy of Sciences and CESS,Tsinghua University
GFDL-CM3<br>GFDL-ESM2G	NOAA Geophysical Fluid Dynamics Laboratory
GISS-E2-H	NASA Goddard Institute for Space Studies
IPSL-CM5A-LR	Institut Pierre-Simon Laplace
MIROC5	Atmosphere and Ocean Research Institute (The University of Tokyo), National Institute for Environmental Studies, and Japan Agency for Marine-Earth Science and Technology
MPI-ESM-MR<br>MPI-ESM-LR	Max-Planck-Institut für Meteorologie (Max Planck Institute for Meteorology)
MRI-CGCM3	Meteorological Research Institute

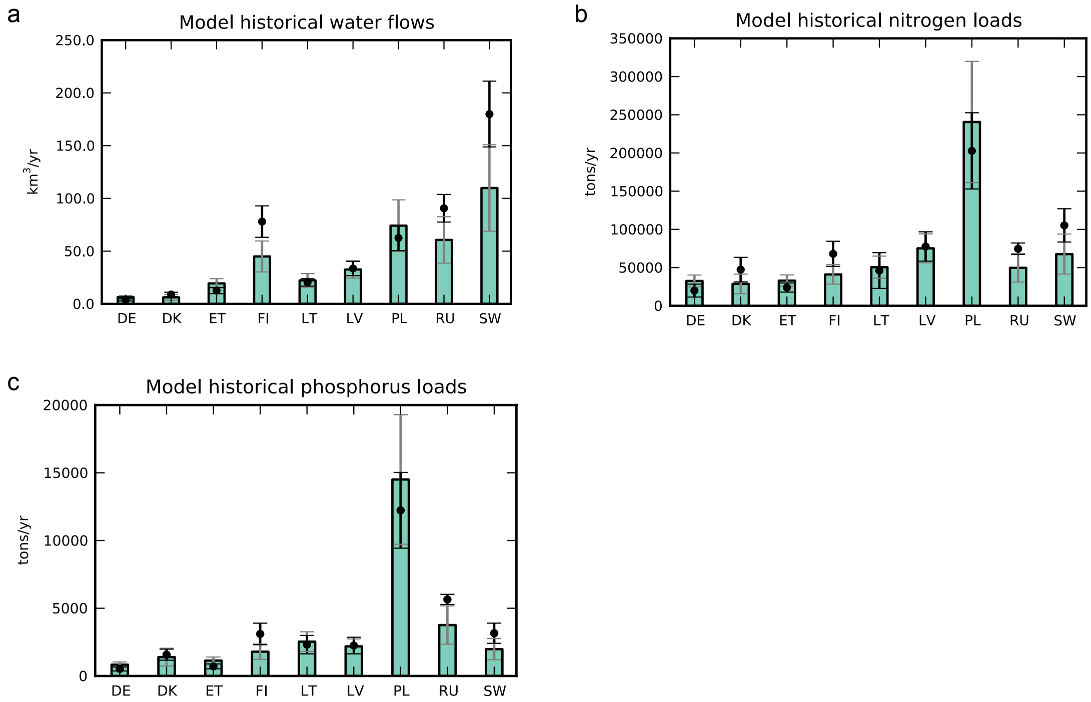

Fig. S1 For the nine countries in the Baltic Sea Drainage Basin (BSDB), bar heights correspond to (a) model historical total water flows, (b) model total riverine nitrogen loads, and (c) model total riverine phosphorus loads. Model values in (b) and (c) are based on reported concentrations in historical runoff. Concentrations were calculated as C = L / Q for averages of annually reported values of loads and total flows for each basin, which were then summed for each country. Circles denote the reported historical values of flows and loads. Note that model values (means for 1961-1990) and reported values (means for 1994-2010) correspond to two different periods. Error bars for model values denote one standard deviation of model means. Error bars for reported values denote one standard deviation of annual means

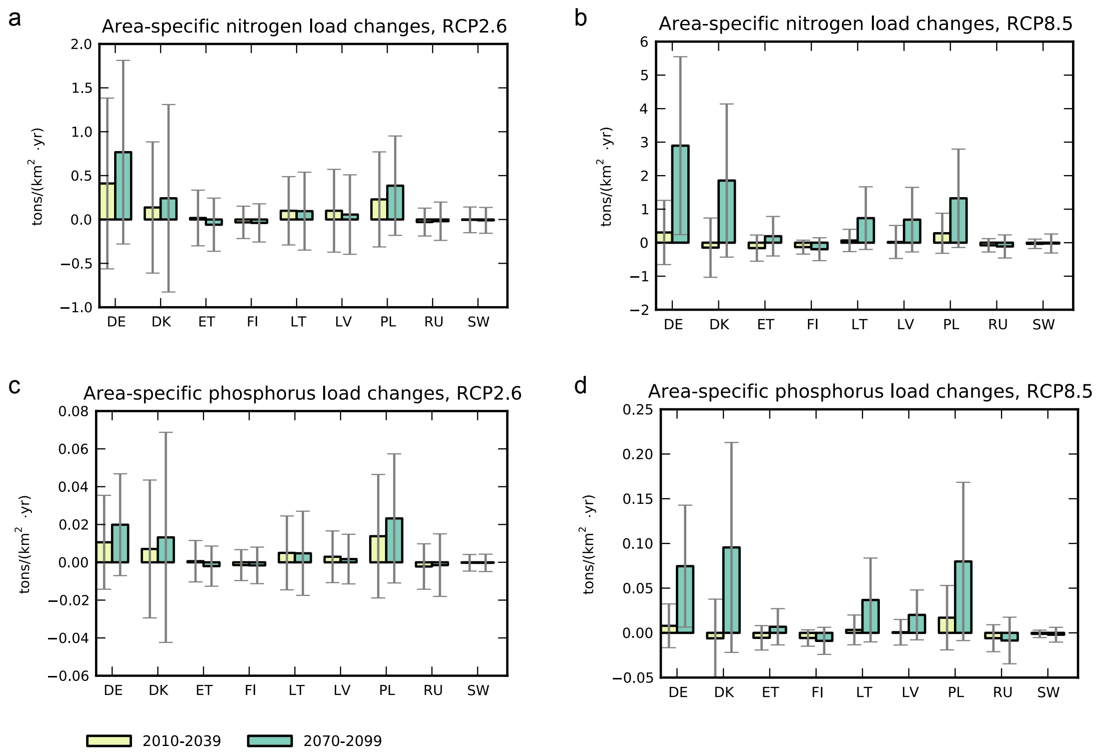

Fig. S2 Mean model (N=13) changes to a-b) area-specific total nitrogen loads, and c-d) area-specific total phosphorus loads for nine countries in the BSDB, from the period 1961-1990 to future periods 2010-2039 and 2070-2099, and for emission scenarios RCP2.6 (a, c) and RCP8.5 (b, d). Error bars denote one standard deviation of model means
